# Distribution of Malocclusion Traits in the Pediatric Population of Milan: An Observational Study

**DOI:** 10.3390/ijerph192114199

**Published:** 2022-10-30

**Authors:** Paolo Caccianiga, Lorenzo Giovanni Mantovani, Marco Baldoni, Gianluigi Caccianiga

**Affiliations:** School of Medicine and Surgery, University of Milano-Bicocca, 20900 Monza, Italy

**Keywords:** oral epidemiology, oral health, malocclusions, orthodontics, diagnostic tools

## Abstract

*Background:* Epidemiological investigations define the prevalence and distribution of the various types of malocclusions, and can help to identify etiological factors and set the most correct orthodontic therapy. *Aim:* The goal of this study was to verify the prevalence and distribution of various types of malocclusions in the pediatric population. *Methods:* The study was performed on a sample of 350 patients (ages 5–9) being treated at the ASST Grande Ospedale Metropolitano Niguarda in Milan. A comparison was presented with one similar epidemiological investigation conducted 22 years earlier by the same researchers. The values of the malocclusion indices were reported from the cephalometric analyzes of the patients and were differentiated on the basis of gender and ethnicity. *Results:* The predominant traits of malocclusion in the general population of the analyzed sample were: skeletal class II (47.43%), hyper-divergence (40.86%), maxillary retrusion (46%), mandibular retrusion (66%), maxillary hypoplasia (50%), mandibular hypoplasia (49.14%), Wits index > 2 mm (22.57%); overjet > 4 mm (31.1%) and overbite > 4 mm (24.86%). Substantial differences were found between Italian patients and patients belonging to different ethnic groups in almost all parameters, and between the male and female genders in some of them. Patients in the 2000 study had a higher prevalence of Class II and hyper-divergence. *Conclusions:* This epidemiological investigation can suggest different approaches in setting the orthodontic treatment plan based on the ethnic group of the patient taken in charge and encourage more specific and large-scale analytical studies on the subject.

## 1. Introduction

In orthodontic clinical practice, it is extremely difficult to observe a perfect and ideal occlusion both at the skeletal and dental level, while it is certainly more frequent to find a “normal” occlusion that is characterized by correct function and good aesthetics, tolerating, therefore small imperfections and irregularities of the bone bases or individual dental elements. Within this biological variability, therefore, there cannot be a clear distinction between a condition that falls within physiological variability and a frankly pathological condition, except perhaps in the case of serious deviations from the norm. Hence, there is a need to codify the skeletal and dental parameters to define a tolerance limit beyond which, in a pathological context, interventions with orthodontic therapy are required.

The study of epidemiological investigations also defines the prevalence and distribution of the various types of malocclusions and can help to identify the etiological factors and set the most correct therapy.

Orthodontic treatment has received a lot of attention from both specialists and patients due to the impact of such treatment on the patient’s social life [1,2]. In clinical practice, many patients are interested in orthodontic treatment, and statistics confirm that malocclusions affect many individuals in the population: 20% of children already suffer from malocclusion at the age of six, and 6% of these patients require an urgent treatment [3].

According to Garattini et al. [4,5] the presence of malocclusions is found in 62–65% of children of pediatric age, while Proffit [6] states that only 44% of the population between six and 16 years has structural and functional alterations to the stomato-gnathic district. In a study by Heikiheimo et al. [7] the data confirm that 20% of six-year-old children are already suffering from malocclusion and in the same age group, 6% of subjects require emergency therapy [8].

Magnusson [9], in a longitudinal study, also found a prevalence of malocclusions of 11% in deciduous dentition and 52% in permanent dentition.

A study conducted in Northern Europe by Evensen and Øgaard [10] confirmed that there has been an increase in the incidence of malocclusions over the last 400–700 years. In the past, malocclusions mainly affected females, while today there is no great difference between the two sexes.

Today we can say that malocclusions do not self-correct with age, but instead tend to get worse. Studies by Heikiheimo et al. [7,11] highlight the need for urgent treatment and how the presence of a relatively severe malocclusion increases from 23% at seven years to 46% at 12 years. Profitt [12] conducted a study on the American population applying the so-called “indicators of the need for orthodontic care”. The study results suggested that 57% to 59% of people of any ethnicity require orthodontic treatment. The same study suggested that the Mexican American population has a higher prevalence of class II malocclusions and class III malocclusions than the rest of the American population, but also a lower prevalence of deep bite and open bite. The most severe cases of malocclusion tend to occur in the African American population.

The objective of the present study was to highlight the prevalence of malocclusions in the Milanese population in an age group between five and nine years and thus to compare the results with the data reported in a similar epidemiological study performed by the same group of researchers from the University of Milan-Bicocca 22 years earlier [13].

## 2. Materials and Methods

This study was conducted on a sample of 350 patients (174 males and 176 females) from the Milanese territory, aged between five and nine years and being treated in the “Paolo Pini” and “Niguarda” dental centers, belonging to the ASST Grande Ospedale Metropolitano Niguarda. All patients underwent a first evaluation visit and were asked to submit the following documentation:

- L-L teleradiography: on this X-ray the cephalometric analysis was performed according to the parameters of the Giannì School (Milan);
- Ortopantomography;
- Study models.
- The following data extrapolated from the cephalometric traces were entered into a database and analyzed:
- Gender;
- Ethnicity;
- Skeletal class: identified through ANB angle analysis;
- Divergence: given by the intermaxillary angle;
- Maxillary position: obtained from the value of the SNA angle;
- Mandibular position: given by the value of the SNB angle;
- Maxillary bone size: given by the value of the SNP-A distance;
- Mandibular size: given by the value of the Go-Me distance;
- Wits index (millimeter distance on the occlusal plane between the orthogonal projections of point A and point B; trend indicator to the III dental class);
- Overjet (horizontal overlap of incisal margins; indicator of II and III skeletal class);
- Overbite (vertical overlap of incisal margins; indicator of deep, normo or open bite);
- Gonial angles (total, upper, lower; growth forecast indicators).

The data were then tabulated, taking as parameters the male and female genders and the ethnic groups most represented in the population sample analyzed: Caucasian (reported as “Italian” as they were all Italian), Arab (patients originating from North Africa or the Middle East), Asian (reported as “Chinese” since they were all of Chinese origin or nationality) and Latin (patients originating from South or Central America).

Patients originating from Romania, Eastern Europe (reported as “Slavs”), sub-Saharan Africa (reported as “African”) and India were taken into consideration only for the analyses reported in the “general population” items, since they were enrolled in a non-sufficient number to perform analyses (the cut-off chosen was 30).

## 3. Results

### 3.1. Gender ed Ethnicity

In the analyzed sample of 350 patients residing in the metropolitan city of Milan treated at the “Paolo Pini” and “Niguarda” odontostomatology centers, a correspondence was found between the number of female (50.29%) and male (49.71%) patients. This correspondence can be found by analyzing the data of Italian and Arab patients; however, it was not found in the data of Chinese and Latin patients.

Of the enrolled patients, 65.7% were of Italian origin. Of the remaining 34.3% of patients of foreign origin, the most represented ethnic groups were: Chinese (10.29%), Latin (9.43%), and Arabs (9.14%) (Table 1).

### 3.2. Skeletal Class

The skeletal class was analyzed with the value of the ANB angle: Class I (0° ≤ ANB ≤ 4°), Class II (ANB > 4°), Class III (ANB < 0°).

The distribution of skeletal classes in the general population is as follows: Class I (49.14%), Class II (47.43%), and Class III (3.43%) (Table 2).

The proportion was roughly respected by dividing the general population by sex, while the distribution of Class III varied, particularly by analyzing the non-Italian ethnic groups, in particular in male patients: 9.38% in Arabs (12.5% in males), 6.06% in Latins (9.52% in males) and 5.56% in Chinese (13.3% in males).

### 3.3. Divergence

The divergence was analyzed with the value of the intermaxillary angle (AJ): Normo-divergence (15° ≤ AJ ≤ 25°), Hypo-divergence (AJ < 15°), and Hyper-divergence (AJ > 25°).

The distribution of divergence in the general population is as follows: Normo-divergence (54.43%), Hypo-divergence (3.71%), Hyper-divergence (40.86%) (Table 3).

Normo-divergence is more frequent in male patients (59.2%) than in females (51.7%), while Hypo-divergence and Hyper-divergence are more frequent in females (4.55% and 43.75%, respectively; against 2.87% and 37.93%).

The proportions were approximately respected considering the Italian and Latin ethnic groups; on the other hand, there was an important increase in Hyper-divergence, especially at the expense of Normo-divergence in the other two ethnic groups analyzed: 61.11% in the Chinese and 59.38% in the Arabs (to note 68.75% in female patients). 

### 3.4. Maxillary Position

The position of the maxillary bone was analyzed with the value of the SNA angle: normo-positioned maxillary bone (80° ≤ SNA ≤ 84°), maxillary retrusion (SNA < 80°), maxillary protrusion (SNA > 84°).

The distribution of the position of the maxillary bone in the general population is as follows: normo-positioned maxillary bone (35.71%), maxillary retrusion (46%), maxillary protrusion (18.29%) (Table 4).

The proportions were quite respected considering the two sexes, with a slight increase in maxillary protrusion in the male sex (20.69% against 15.9%) and considering the Italian and Arab patients (where there was an important difference in the protrusion maxillary in males: 37.5% versus 6.25% in females).

There are important differences to take into consideration the other two ethnic groups analyzed: in the Chinese there is a normal positioned jaw in 63.89% of cases, a maxillary retrusion in 22.22% and a maxillary protrusion in 13.89%, with no particular differences between males and females; while in the Latins the aforementioned values are respectively, 36.36%, 24.24% and 39.39%, with a higher prevalence of a normal-positioned jaw in females (50%) and of a maxillary protrusion in males (47.62%).

### 3.5. Mandibular Position

The position of the mandible was analyzed with the value of the SNB angle: normo-positioned mandible (78° ≤ SNB ≤ 82°), mandibular retrusion (SNB < 78°), and mandibular protrusion (SNB > 82°).

The distribution of the position of the mandible in the general population is as follows: normo-positioned mandible (24.57%), mandibular retrusion (66%), mandibular protrusion (9.71%) (Table 5).

The proportions were quite respected considering the two sexes and the Italian, Arab and Chinese patients. As for Latin patients, there was an increase in mandibular protrusion (21.21%) and a decrease in mandibular retrusion (54.55%). Note the difference between the values in the two sexes in the ethnic group of Arab patients: in males the mandibular normo-position was 50%, the mandibular retrusion 43.75% and the mandibular protrusion 6.25%; while in females the above-mentioned values were, respectively, 12.5%, 75% and 12.5%.

### 3.6. Maxillary Bone Size

Maxillary bone size was analyzed with SNP-A distance value: normal sized maxilla (41.8 mm ≤ SNP-A ≤ 45.8 mm), maxillary hypoplasia (SNP-A < 41.8 mm), maxillary hyperplasia (SNP-A > 45.8 mm).

The distribution of the position of the jawbone in the general population is as follows: normal sized maxilla (40%), maxillary hypoplasia (50%), maxillary hyperplasia (10%) (Table 6).

The values of maxillary hyperplasia remained approximately unchanged considering the two sexes and the different ethnic groups, with the exception of the Arab patients in which it was increased (15.6%).

There was a greater tendency to maxillary hypoplasia in females (54.55%) and normal sized maxilla in males (45.4%); characteristic of what is found in Italian patients (females, maxillary hypoplasia: 59.48%; males, normal sized maxilla: 51.75%) and Latin patients (females, maxillary hypoplasia: 58.33%; males, normal sized maxilla: 47.62%).

In Arab female patients there was a reversal of this trend: a greater distribution of normal sized maxilla (50%) and a lower distribution of maxillary hypoplasia (31.25%).

In Chinese male patients we found a low number of cases of normo-maxilla (20%) and a high number of cases of maxillary hypoplasia (80%).

### 3.7. Mandible Size

The size of the mandibular bone is analyzed with the Go-Me distance value: normal sized mandible (59.7 mm ≤ Go-Me ≤ 63.7 mm), mandibular hypoplasia (Go-Me < 59.7 mm), mandibular hyperplasia (Go-Me > 63.7 mm).

The distribution of the position of the maxillary bone in the general population is as follows: normal sized mandible (17.71%), mandibular hypoplasia (49.14%), mandibular hyperplasia (33.14%) (Table 7).

The proportions were approximately respected considering the two sexes and the Italian, Arab and Latin patients. On the other hand, considering Chinese patients, there was an important increase in the distribution of mandibular hyperplasia (44.44%), represented above all in female patients (57.14%), while in male patients a situation of mandibular hypoplasia was predominant (66.67%).

### 3.8. Wits Index

The Wits index is a millimeter distance on the occlusal plane between the orthogonal projections of point A and point B. It is an indicator of a trend towards dental class III in the event of a strongly negative value. The normal range is between −2 mm and 2 mm.

The distribution of the Wits Index in the general population is as follows: −2 mm ≤ W ≤ 2 mm (57.13%), W < −2 mm (20.29%), W > 2 mm (22.57%) (Table 8).

The proportions are approximately respected considering the two sexes and the Italian and Chinese patients. In Arab patients there was a greater distribution of a Wits Index <−2 mm (34.38%), especially in male patients (43.75%); the same was found in Latin patients (30.3%), especially in female patients (41.67%). It follows that Arab and Latin patients had a greater tendency to the third dental class.

### 3.9. Overjet

The overjet is the horizontal overlap of the incisal margins and is an indicator of II and III skeletal class. The normal range is between 0.5 mm and 5 mm.

The distribution of overjet in the general population is as follows: 0.5 mm ≤ OJ ≤ 4 mm (60.86%), OJ < 0.5 mm (8%), OJ > 4 mm (22.57%) (Table 9).

Considering the two sexes, there is a greater tendency for females to have an overjet between 0.5 and 4 mm (67.05%) and a greater tendency for males to have an overjet greater than 4 mm (37.9%).

The same proportion was found in Italian patients. In Arab patients there was a greater distribution of patients with an overjet of less than 0.5 mm (15.6%); in Chinese and Latin patients there was a greater distribution of overjet greater than 4 mm, with percentages of 41.7% and 42.4%, respectively. Furthermore, in Chinese patients there was an important variability between the two sexes: in males the overjet values greater than 4 mm (60%) were higher, while in female patients the overjet values were lower than 0.5 mm (23.8%).

### 3.10. Overbite

The overbite is the vertical overlap of the incisal margins and is an indicator of deep, normo or open bite. The normal range is between 0.5 mm and 5 mm.

The distribution of overbite in the general population is as follows: 0.5 mm ≤ OJ ≤ 4 mm (59.14%), OJ < 0.5 mm (16%), OJ > 4 mm (24.86%) (Table 10).

There are important differences between the two sexes: in males overbite values greater than 4 mm are more frequent (29.31%), a greater tendency to deep bite, while in females there are normal values (64.2%).

The same proportions were approximately respected in Italian and Chinese patients. In Arab patients, there was an increase in overbite values greater than 4 mm (34.38%), while in Latin patients the distribution of these values was decreased (15.15%).

The distribution of overbite values of less than 0.5 mm, and therefore the tendency to open bite, were fairly constant regardless of gender and ethnic groups.

### 3.11. Total Gonial Angle

The gonial angles are indicators of growth forecast. We distinguish total gonial angle, upper gonial angle and lower gonial angle.

The indicated “normal” range, even if the patients examined are of different ages and between 5 and 9 years and therefore in different stages of growth, for the total gonial angle is between 115° and 125°.

The distribution of the total gonial angle values in the general population is as follows: 115° ≤ GON ≤ 125° (33.43%), GON < 115° (2.57%), GON > 125° (64%) (Table 11).

In females, the number of values in the “normal” range was increased (36.36%). For the rest, the proportions were approximately respected considering the two sexes and the Italian and Latin patients. In Arab patients there was an increase in patients with a total gonial angle greater than 125° (75%) and a decrease in patients with a total gonial angle in “normal” range (21.88%). In Chinese patients, on the other hand, there was a decrease in patients with a gonial angle greater than 125° (55.56%).

### 3.12. Upper Gonial Angle

The indicated “normal” range, even if the patients examined are of different ages and between 5 and 9 years and therefore in different stages of growth, for the upper gonial angle is between 48° and 52°.

The distribution of the upper gonial angle values in the general population is as follows: 48° ≤ GS ≤ 52° (17.43%), GS < 48° (5.14%), GS > 52° (77.43%) (Table 12).

The proportions were approximately respected considering the two sexes and the Italian and Arab patients. In Chinese patients, the number of values in the “normal” range was increased (30.56%) and the number of upper gonial angle values below 48° (19.4%); on the other hand, the number of patients with an upper gonial angle greater than 52° decreased (50%). In Latin patients there was a significant decrease in patients with GS > 52° (63.64%).

### 3.13. Lower Gonial Angle

The indicated “normal” range, even if the patients examined are of different ages and between five and nine years and therefore in different stages of growth, for the lower gonial angle is between 67° and 73°.

The distribution of the lower gonial angle values in the general population is as follows: 67° ≤ GI ≤ 73° (47.14%), GS < 67° (12%), GS > 73° (40.86%) (Table 13).

The proportions were approximately respected considering the two sexes. However, the values changed significantly considering the ethnic groups: the values of the Italian patients were, respectively, 50.87%, 15.65% and 33.48%; the values of Arab patients were, respectively, 28.13%, 6.25% and 65.63%; the values of Chinese patients were, respectively, 33.33%, 2.78% and 63.89%; the values of Latin patients were, respectively, 54.55%, 6.06% and 39.39%.

### 3.14. Comparative Analysis between the Data Obtained from the Epidemiological Survey of the Present Study and Those Obtained in the Similar Study of 2000 [13]

Table 14 shows a greater differentiation of the ethnicity of the sample in the 2022 study, compared to the previous one.

The parameters analyzed common to the two studies were: skeletal classes and divergence.

These above-mentioned parameters were analyzed with reference to the values of the general population.

## 4. Discussion

In the literature, there is a wide heterogeneity of epidemiological studies on the distribution of malocclusion traits in the pediatric population, especially on the distribution of skeletal classes, performed on population samples of very different ethnicities and in the number of patients enrolled.

In a study by Albini Riccioli [14], conducted in Pesaro, occlusal alterations were present in 40.6%: these malocclusions were represented by Class II in 37.7% of cases and by Class III in 2.9%.

In a study by Parker [15], in which 1000 patients were enrolled, aged between 9 and 21 years, 42% presented malocclusions, divided into 35% of Class II and 7% of Classes III.

Siriwat and Jarabak [16] found that the most frequent malocclusions were Class I (47%), followed by Class II (46.4%) and Class III (6.6%).

Also similar are the results of a study conducted in Jerusalem [17] on 989 children, aged between six and 13: Class I malocclusions were present in 49.1% of subjects, Class II in malocclusions were present in 46.2% of subjects, and 0.7% had Class III malocculasions.

Significantly different results emerged from a study of 108 young adults in Hong Kong [18], which showed that 58.4% had normal occlusion or in any case did not require orthodontic intervention, 21.3% had Class II malocclusions, and as many as 14.8% had Class III malocclusions, data significantly higher than European or American statistics.

An epidemiological study conducted in Nigeria by Aikins et al. [19] on 320 patients aged between 13 and 20 years, found that 11.8% of patients had normal occlusion, 3% had a class I malocclusion, 6.3% had a class II malocclusion and 1.6% had a class III malocclusion.

Similar results were found in Italy [20] in a study conducted on 3017 patients aged between eight and 13 years. It was found that 75.8% of patients had malocclusions accompanied by widespread premature loss of deciduous teeth and increased overjet and overbite.

Aldrees [21] conducted a study in Saudi Arabia also confirming that the most common malocclusions were those of class I.

The same results were found in a study conducted in China [22]: once again the most common cases of malocclusion were class I (48%), but what differentiated the results obtained in this study from those obtained in European studies and Americans was the predominance of class III malocclusions (21%).

In a recent systematic review of the literature, with the aim of determining the distribution of malocclusion traits throughout the world in mixed and permanent dentitions, Alhammadi et al. [23], reported that in the permanent dentition, the global distributions of Class I, Class II and Class III malocclusions were respectively 74.7%, 19.56% and 5.93%; in mixed dentition, the distribution of these malocclusions was 73%, 23% and 4%. For vertical malocclusions, deep bite and open bite malocclusions were 21.98% and 4.93%, respectively. Africans showed the highest prevalence of Class I and open bite in permanent dentition (89% and 8%, respectively), and in mixed dentition (93% and 10%, respectively), while Caucasians showed the highest prevalence of Class II in permanent dentition (23%) and mixed dentition (26%). Class III malocclusion in mixed dentition was highly prevalent among Asians.

Among the factors that can be identified as the cause of this wide variability in the literature, there is undoubtedly the diversity of statistical methods used and the samples of subjects examined.

Substantial differences can also be found in the distributions of skeletal classes and divergence between the study conducted in 2000 by Caccianiga et al. [13] (Table 15 and Table 16) and our 2022 study.

In the 2000 study [13], only patients of Italian origin were enrolled, while in the 2022 study more than one third of the patients enrolled were of foreign origin, which made it possible to differentiate the parameters analyzed according to the different ethnic groups they belonged to. Substantial differences were found between Italian patients and patients belonging to different ethnic groups in the parameters: skeletal class (class III most represented in Arabs, Latin and Chinese), divergence (hyper-divergence more represented in Chinese and Arabs), maxillary position (normo-position more represented in the Chinese and protrusion in the Latins), mandibular position (protrusion more represented in the Chinese), maxillary bone size (maxillary hypoplasia more represented in the Chinese), mandibular size (mandibular hyperplasia more represented in the Chinese), Wits index (values of W < −2 mm more represented in Arabic and Latin), overjet and overbite (overjet increased in Chinese and Latin and decreased in Arabs; overbite increased in Arabs) and gonial angles (GON increased in Arabs and decreased in Chinese; GS decreased in Chinese and Latin; GI increased in Arabs and Chinese).

## 5. Conclusions

In conclusion, this study appears to be useful to the scientific literature and to the orthodontic clinic since it presents the distribution of different indices of malocclusion, in a higher number than most similar studies. Within the same population sample were highlighted some differences in the distribution of malocclusions in the different ethnic groups analyzed in the present study.

From the comparison with the 2000 study carried out on the same territory, the difference of some parameters was highlighted. In particular, a decrease in the prevalence of class II skeletal malocclusion and hyper-divergence was noted. A possible explanation could be the greater attention paid by families to the malocclusions of their children in recent years compared to 2000, thanks to the numerous interventions to raise awareness of orthodontic problems, which would explain why patients with “less severe” forms of malocclusion have come to our attention compared to patients 22 years ago.

Even within its limits, linked to the low number of patients not of Italian origin and to its being only a descriptive study, this epidemiological investigation may suggest the need to develop different therapy plans in the orthodontic field on the basis of the patient’s ethnicity, since the current parameters defined in the physiological range have been defined based on a predominantly Caucasian population. A certain parameter that can be pathological according to traditional clinical cephalometric methods could instead be physiological for a patient of non-Caucasian ethnicity.

Therefore, this can be considered a “preliminary study” to more specific and large-scale epidemiological investigations in which analyze these differences, and which can lead to the differentiation of therapeutic plans on the basis of the ethnic factor.

## Figures and Tables

**Table 1:** Distribution of ethnicity and gender in the selected patient sample (in bold the most represented ethnic groups).

	Total	% on Total	M	% Relative	F	% Relative
**Italian**	230	**65.7**	114	49.57	116	50.43
Romanian	2	0.57	1	50	1	50
Slavs	9	2.57	5	55.56	4	44.44
**Arab**	32	**9.14**	16	50	16	50
African	5	1.43	2	40	3	60
Indian	3	0.86	0	0	3	100
**Chinese**	36	**10.29**	15	41.67	21	58.33
**Latin**	33	**9.43**	21	63.64	12	36.36
Total	350	100	174	49.71	176	50.29

**Table 2:** Distribution of skeletal classes in the selected patient sample.

	Class I (0° ≤ ANB ≤ 4°)	%	Class II (ANB > 4°)	%	Class III (ANB < 0°)	%
**General population**	172	49.14	166	47.43	12	3.43
M	85	48.85	82	47.13	7	4.02
F	87	49.43	84	47.73	5	2.84
**Italian**	118	49.37	107	46.52	5	2.17
M	65	57.02	48	42.11	1	0.88
F	53	45.69	59	50.86	4	3.45
**Arab**	14	43.75	15	46.88	3	9.38
M	6	37.5	8	50	2	12.5
F	8	50	7	43.75	1	6.25
**Chinese**	18	50	16	44.44	2	5.56
M	3	20	10	66.67	2	13.3
F	15	71.43	6	28.57	0	0
**Latin**	14	42.42	17	51.52	2	6.06
M	8	38.1	11	52.38	2	9.52
F	6	50	6	50	0	0

**Table 3:** Distribution of divergence in the selected patient sample.

	Normo Div. (15° ≤ AJ ≤ 25°)	%	Hypo Div. (AJ < 15°)	%	Hyper Div. (AJ > 25°)	%
**Gen. pop.**	194	55.43	13	3.71	143	40.86
M	103	59.2	5	2.87	66	37.93
F	91	51.7	8	4.55	77	43.75
**Italian**	139	60.43	10	4.35	81	35.22
M	72	63.16	4	3.51	38	33.33
F	67	57.76	6	5.17	43	37.07
**Arab**	13	40.63	0	0	19	59.38
M	8	50	0	0	8	50
F	5	31.25	0	0	11	68.75
**Chinese**	14	38.89	0	0	22	61.11
M	6	40	0	0	9	60
F	8	38.1	0	0	13	61.9
**Latin**	17	51.52	2	6.06	14	42.42
M	10	47.62	1	4.76	10	47.62
F	7	58.33	1	8.33	4	33.33

**Table 4:** Distribution of the position of the maxillary bone in the selected patient sample.

	Normo Max. Position (80° ≤ SNA ≤ 84°)	%	Max. Retrusion (SNA < 80°)	%	Max. Protrusion (SNA > 84°)	%
**Gen. pop.**	125	35.71	161	46	64	18.29
M	61	35.06	77	44.25	36	20.69
F	64	36.36	84	47.73	28	15.9
**Italian**	94	43.04	104	45.22	32	13.91
M	39	34.21	60	52.63	15	13.16
F	55	47.41	44	37.93	17	14.66
**Arab**	11	34.38	14	43.75	7	21.87
M	4	35	6	37.5	6	37.5
F	7	43.75	8	50	1	6.25
**Chinese**	23	63.89	8	22.22	5	13.89
M	10	66.67	3	20	2	13.33
F	13	61.9	5	23.81	3	14.29
**Latin**	12	36.36	8	24.24	13	39.39
M	6	28.57	5	32.81	10	47.62
F	6	50	3	25	3	25

**Table 5:** Distribution of mandibular position in the selected patient sample.

	Normo Man. Position (78° ≤ SNB ≤ 82°)	%	Man. Retrusion (SNB < 78°)	%	Man. Protrusion (SNB > 82°)	%
**Gen. pop.**	86	24.57	231	66	34	9.71
M	44	25.29	113	64.94	17	9.77
F	42	23.86	117	66.48	17	9.66
**Italian**	55	23.91	159	69.13	16	6.96
M	25	21.93	81	71.05	8	7.02
F	30	25.86	78	67.24	8	6.9
**Arab**	10	31.25	19	59.38	3	9.4
M	8	50	7	43.75	1	6.25
F	2	12.5	12	75	2	12.5
**Chinese**	9	25	22	61.11	4	11.11
M	4	26.67	9	60	2	13.33
F	5	23.81	13	61.9	2	9.52
**Latin**	8	24.24	18	54.55	7	21.21
M	5	23.81	11	52.38	5	23.81
F	3	25	7	58.33	2	16.67

**Table 6:** Distribution of maxillary bone size values in the selected patient sample.

	Normal Sized Maxilla (41.8 mm ≤ SNP-A ≤ 45.8 mm)	%	Maxillary Hypoplasia (SNP-A < 41.8 mm)	%	Maxillary Hyperplasia (SNP-A > 45.8 mm)	%
**Gen. pop.**	140	40	175	50	35	10
M	79	45.4	79	45.4	16	9.2
F	61	34.66	96	54.55	19	10.8
**Italian**	97	42.17	114	49.57	19	8.26
M	59	51.75	45	39.47	10	8.77
F	38	32.76	69	59.48	9	7.76
**Arab**	14	43.75	13	40.63	5	15.6
M	6	37.5	8	50	2	12.5
F	8	50	5	31.25	3	18.8
**Chinese**	11	30.56	21	58.33	4	11.1
M	3	20	12	80	0	0
F	8	38.1	9	42.86	4	19
**Latin**	13	39.39	17	51.52	3	9.09
M	10	47.62	10	47.62	1	4.76
F	3	25	7	58.33	2	16.7

**Table 7:** Distribution of mandible size values in the selected patient sample.

	Normal Sized Mandible (59.7 mm ≤ Go-Me ≤ 63.7 mm)	%	Mandibular Hypoplasia (Go-Me < 59.7 mm)	%	Mandibular Hyperplasia (Go-Me > 63.7 mm)	%
**Gen. pop.**	62	17.71	172	49.14	116	33.14
M	31	17.82	89	51.15	54	31.03
F	31	17.61	83	47.16	62	35.23
**Italian**	43	18.7	116	50.43	71	30.87
M	23	20.18	55	48.25	36	31.58
F	20	17.24	61	52.59	35	30.17
**Arab**	8	25	12	37.5	12	37.5
M	4	25	7	43.75	5	31.25
F	4	25	5	31.25	7	43.75
**Chinese**	3	8.33	17	47.22	16	44.44
M	1	6.67	10	66.67	4	26.67
F	2	9.52	7	33.33	12	57.14
**Latin**	5	15.15	15	45.45	13	39.39
M	2	9.52	11	52.38	8	38.1
F	3	25	4	33.33	5	41.67

**Table 8:** Distribution of the Wits Index values in the selected patient sample.

	−2 mm ≤ W ≤ 2 mm	%	W < −2 mm	%	W > 2 mm	%
**Gen. pop.**	200	57.14	71	20.29	79	22.57
M	96	55.17	34	19.54	44	25.29
F	104	59.09	37	21.02	35	19.89
**Italian**	136	59.13	37	16.09	57	24.78
M	69	60.53	18	15.79	27	23.68
F	67	57.76	19	16.38	30	25.86
**Arab**	16	50	11	34.38	5	15.63
M	5	31.25	7	43.75	4	25
F	11	68.75	4	25	1	6.25
**Chinese**	20	55.56	8	22.22	8	22.22
M	8	53.33	3	20	4	26.67
F	12	57.14	5	23.81	4	19.05
**Latin**	16	48.48	10	30.3	7	21.21
M	10	47.62	5	23.81	6	28.57
F	6	50	5	41.67	1	8.333

**Table 9:** Distribution of overjet values in the selected patient sample.

	0.5mm ≤ OJ ≤ 4 mm	%	OJ < 0.5 mm	%	OJ > 4 mm	%
**Gen. pop.**	213	60.86	28	8	109	31.1
M	95	54.6	13	7.47	66	37.9
F	118	67.05	15	8.52	43	24.4
**Italian**	148	64.35	14	6.09	68	29.6
M	67	58.77	8	7.02	39	34.2
F	81	69.83	6	5.17	29	25
**Arab**	21	65.63	5	15.6	6	18.8
M	9	56.25	3	18.8	4	25
F	12	50	2	12.5	2	12.5
**Chinese**	16	44.44	5	13.9	15	41.7
M	6	40	0	0	9	60
F	10	47.62	5	23.8	6	28.6
**Latin**	16	48.48	3	9.09	14	42.4
M	10	47.62	1	4.76	10	47.6
F	6	50	2	16.7	4	33.3

**Table 10:** Distribution of overbite values in the selected patient sample.

	0.5mm ≤ OB ≤ 4 mm	%	OB < 0.5 mm	%	OB > 4 mm	%
**Gen. pop.**	207	59.14	56	16	87	24.86
M	94	54.02	29	16.7	51	29.31
F	113	64.2	27	15.3	36	20.45
**Italian**	137	59.57	36	15.7	57	24.78
M	60	52.63	22	19.3	32	28.07
F	77	66.38	14	12.1	25	21.55
**Arab**	17	53.13	4	12.5	11	34.38
M	9	56.25	2	12.5	5	31.25
F	8	50	2	12.5	6	37.5
**Chinese**	20	55.56	7	19.4	9	25
M	7	46.67	2	13.3	6	40
F	13	61.9	5	23.8	3	14.29
**Latin**	22	66.67	6	18.2	5	15.15
M	14	66.67	2	9.52	5	23.81
F	8	66.67	4	33.3	0	0

**Table 11:** Distribution of the total gonial angle values in the selected patient sample.

	115° ≤ GON ≤ 125°	%	GON < 115°	%	GON > 125°	%
**Gen. pop.**	117	33.43	9	2.57	224	64
M	53	30.46	5	2.87	116	66.67
F	64	36.36	4	2.27	108	61.36
**Italian**	78	33.91	5	2.17	147	63.91
M	37	32.46	2	1.75	75	65.79
F	41	35.34	3	2.59	72	62.07
**Arab**	7	21.88	1	3.13	24	75
M	3	18.75	1	6.25	12	75
F	4	25	0	0	12	75
**Chinese**	13	36.11	3	8.33	20	55.56
M	5	33.33	2	13.3	8	53.33
F	8	38.1	1	4.76	12	57.14
**Latin**	12	36.36	0	0	21	63.64
M	7	33.33	0	0	14	66.67
F	5	41.67	0	0	7	58.33

**Table 12:** Distribution of the upper gonial angle values in the selected patient sample.

	48° ≤ GS ≤ 52°	%	GS < 48°	%	GS > 52°	%
**Gen. pop.**	61	17.43	18	5.14	271	77.43
M	32	18.39	6	3.45	136	78.16
F	29	16.48	12	6.82	135	76.7
**Italian**	34	14.78	6	2.61	190	82.61
M	20	17.54	1	0.88	93	81.58
F	14	12.07	5	4.31	97	83.62
**Arab**	5	15.63	2	6.25	25	78.13
M	1	6.25	1	6.25	14	87.5
F	4	25	1	6.25	11	68.75
**Chinese**	11	30.56	7	19.4	18	50
M	4	26.67	3	20	8	53.33
F	7	33.33	4	19	10	47.62
**Latin**	9	27.27	3	9.09	21	63.64
M	7	33.33	1	4.76	13	61.9
F	2	16.67	2	16.7	8	66.67

**Table 13:** Distribution of the lower gonial angle values in the selected patient sample.

	67° ≤ GI ≤ 73°	%	GI < 67°	%	GI > 73°	%
**Gen. pop.**	165	47.14	42	12	143	40.86
M	85	48.85	19	10.92	70	40.23
F	80	45.45	23	13.07	73	41.48
**Italian**	117	50.87	36	15.65	77	33.48
M	59	51.75	16	14.04	39	34.21
F	58	50	20	17.24	38	32.76
**Arab**	9	28.13	2	6.25	21	65.63
M	6	37.5	1	6.25	9	56.25
F	3	18.75	1	6.25	12	75
**Chinese**	12	33.33	1	2.78	23	63.89
M	6	40	0	0	9	60
F	6	28.57	1	4.76	14	66.67
**Latin**	18	54.55	2	6.06	13	39.39
M	11	52.38	1	4.73	9	42.86
F	7	58.33	1	8.33	4	33.33

**Table 14:** Comparison of the number and ethnic groups of patients enrolled in the epidemiological studies of 2000 and 2022.

	N° Patients Enrolled	Most Represented Ethnic Groups
**2000 study**	342	Italian (100%)
**2022 study**	350 (174 M; 176 F)	Italian (65.7%), Chinese (10.29%), Latin (9.43%), Arab (9.14%)

**Table 15:** Comparison of the distribution of skeletal classes in patients enrolled in the epidemiological studies of 2000 and 2022.

	Class I	Class II	Class III
**2000 study**	41.2%	56.2%	2.6%
**2022 study**	49.14%	47.43%	3.43%

Class II is markedly more represented in the 2000 study (56.2% versus 44.9% and 47.43%) and less so for Classes I (41.2 % versus 49.21% and 49.14%) and III (2.6% versus 5.9% and 3.43%).

**Table 16:** Comparison of the distribution of divergence in patients enrolled in the epidemiological studies of 2000 and 2022.

	Normo-Divergence	Hypo-Divergence	Hyper-Divergence
**2000 study**	27.8%	0.6%	71.7%
**2022 study**	55.43%	4.71%	40.86%

In the 2000 study the hyper-divergence is markedly more represented (71.7% versus 39.76% and 40.86%) and less so for the normo-divergence (27.8% versus 51.71% and 55.43%) and the hypo-divergence (0.6% vs. 8.54% and 4.71%).
